# Establishment of an experimental pig model for the induction of a *Staphylococcus hyicus* skin infection

**DOI:** 10.17221/68/2025-VETMED

**Published:** 2026-03-30

**Authors:** Katarina Matiaskova, Marketa Reichelova, Edita Jeklova, Monika Zouharova, Sarka Kobzova, Katerina Nedbalcova, Jan Matiasovic, Martin Faldyna

**Affiliations:** ^1^Department of Infectious Diseases and Preventive Medicine, Veterinary Research Institute, Brno, Czech Republic; ^2^Department of Microbiology and Antimicrobial Resistance, Veterinary Research Institute, Brno, Czech Republic

**Keywords:** bacteria, exudative epidermitis, Staphylococcaceae, weaning, wounds

## Abstract

*Staphylococcus hyicus* is one of the causative agents of exudative epidermitis in pigs. The aim of this study was to establish a porcine challenge model of a skin infection caused by *S. hyicus* to assess the effectiveness of a medicinal product intended for local application. Based on the results of the presence of toxin encoding genes and antimicrobial resistance (detected resistance to clindamycin, penicillin, ampicillin, erythromycin and tetracycline), three field strains were selected for the trial. At D0, six surface defects were created on the back of six piglets. The defects were inoculated with bacteria at two different concentrations: 1 × 10^8^ or 1 × 10^9 ^CFU/ml. Every day throughout the experiment, the pigs were monitored, and their rectal temperatures were measured. On D4, D9, and D14, a visual evaluation and indirect bacteriological imprints of the defects were performed. Crusts were present from D9, and a lower bacterial concentration led to reduced secretion and crust formation. Based on the results of bacterial cultivation of the indirect imprints, however, *S. hyicus* was present in greater amounts in the wound defects infected with the lower bacterial concentration. As there were differences in the obtained results among the strains used, it can be concluded that the strain marked as CAPM 6689 seems to be the most applicable and the lower concentration was enough for the infection development.

Exudative epidermitis, also known as greasy pig disease, is a common bacterial skin disease of swine worldwide. The clinical picture in piglets between 7 and 28 days covers generalised non-pruritic exudative epidermitis. In older animals, a mainly localised form affecting the head and extremities occurs (Frana and Hau 2019).

The main causative agent associated with exudative epidermitis is *Staphylococcus hyicus*. This Gram-positive coccus is coagulase-variable, catalase-positive, and non-haemolytic on blood agar, forming non-pigmented, opaque colonies with entire margins (Schleifer and Bell 2009). Most strains ferment many sugars, among others fructose, glucose, and lactose (Lammler 1991).

A crucial virulence factor is the production of exfoliative toxins (Andresen et al. 1993; Wegener et al. 1993; Tanabe et al. 1996). All the toxigenic strains of *S.* *hyicus* encode at least one of the exfoliative toxins – ExhA, ExhB, ExhC, or ExhD (Leekitcharoenphon et al. 2016). These toxins are similar to the exfoliative toxins from *Staphylococcus aureus* and together constitute a homologous family (Ahrens and Andresen 2004). The toxins have been shown to cleave desmoglein-1 (Fudaba et al. 2005; Nishifuji et al. 2005), a structural protein in the epidermis that helps maintain tissue structure through its adhesive function (Hammers and Stanley 2013).

Although some authors have described the successful control of the disease outbreak using an autogenous vaccine (Arsenakis et al. 2018; Romero-Salmoral et al. 2025), there is no specific commercial vaccine for the active immunisation of piglets. The treatment of the disease involves the use of antibiotics. However, a number of studies have demonstrated increasing trends in antibiotic-resistant strains of *S. hyicus* in swine herds worldwide (Aarestrup and Jensen 2002; Park et al. 2013a; Park et al. 2013b; Moreno et al. 2022; Ocloo et al. 2022; Mencia-Ares et al. 2024). Therefore, there is a strong need to find other, non-antibiotic solutions. Plant based biologically active compounds are one of the promising ways to address this situation. For example, plant essential oils and polyphenols have been shown, in many studies, to have antibacterial effects, including mechanisms that damage the bacterial membrane or suppress biofilm formation (Miklasinska-Majdanik et al. 2018; Vaillancourt et al. 2018).

Therefore, the aim of the study was to establish a porcine challenge model of a skin infection with *S. hyicus* to assess the effectiveness of an alternative medicinal product intended for local application to the infected skin.

## MATERIAL AND METHODS

### Bacterial isolates and growth conditions

A total of five *S.* *hyicus* strains from pigs were used in this study. The historical strain BEZ (CAPM 6346) was obtained from the Collection of Animal Pathogenic Microorganisms (CAPM) of the Veterinary Research Institute (VRI). Four strains (STH 1/22, STH 2/22, STH 3/22 and STH 4/22) were isolated from clinical cases of exudative epidermitis in three pig farms in the Czech Republic in 2022 before the start of any treatment. Skin swabs were inoculated onto Baird-Parker agar (Oxoid Ltd., Basingstoke, United Kingdom) and blood agar base No. 2 (Oxoid Ltd., Basingstoke, United Kingdom) with 7% defibrinated sheep blood (LabMediaServis, Jaroměř, Czech Republic) and incubated for 18–24 h at 37 °C under aerobic conditions. Suspected *S.* *hyicus* colonies were selected and their identity was verified by matrix-assisted laser desorption-ionisation time-of-flight (MALDI-TOF) mass spectrometry (MS), whole genome sequencing (WGS) and commercial biochemical systems for staphylococci. After identification, the four above-mentioned *S.* *hyicus* strains were deposited in CAPM under the numbers CAPM 6689, CAPM 6690, CAPM 6691, and CAPM 6692. *S.* *hyicus* CAPM 6346 was grown on blood agar for 18–24 h at 37 °C and evaluated by the same methods.

### MALDI-TOF mass spectrometry (MS) identification

MALDI-TOF MS was used for the confirmation of the *S.* *hyicus* strains. The samples were prepared using (1) the ethanol-formic acid extraction procedure recommended by Bruker Daltonics and (2) the modified formic acid extraction method (Zhu et al. 2015). For each analysed sample, extraction mixtures were spotted in duplicate onto a MALDI target plate. The bacterial strains were identified by comparing the mass spectra against the Bruker MBT 8468 database in the Biotyper software (v3.1.66; Bruker Daltonics GmbH, Bremen, Germany).

### Biochemical characterisation

All five strains were evaluated using the commercial biochemical identification kits STAPHYtest 24 (Erba Lachema, Brno, Czech Republic) and API^®^ Staph (BioMérieux SA, Marcy-l'Étoile, France). The test results were read manually. Some additional tests were performed: coagulase test with rabbit coagulase plasma for the detection of the bound (clumping factor) and free coagulase (Pro-Lab Diagnostics, Inc., Bromborough, United Kingdom), novobiocin resistance with diagnostic disc NOVOBIOCIN (Erba Lachema, Brno, Czech Republic), VP test for the detection of acetoin production (Erba Lachema, Brno, Czech Republic), PYRAtest for the detection of the pyrrolidonyl arylamidase (PYR) activity (Erba Lachema, Brno, Czech Republic), OXItest for the detection of cytochrome oxidase (Erba Lachema, Brno, Czech Republic), catalase test (BioMérieux SA, Marcy-l'Étoile, France) and pigment production on Tryptone Soya Agar (Oxoid Ltd., Basingstoke, United Kingdom). The tests were performed according to the manufacturer’s instructions. The evaluation of the obtained results was performed with the software ErbaExpert Vet (v1.0.03; Erba Lachema, Brno, Czech Republic) or using the online identification software apiwebTM (https://apiweb.biomerieux.com/login).

### Whole genome sequencing

Genomic DNA from the isolates was isolated using the Qiagen DNeasy Blood and Tissue Kit (QIAGEN GmbH, Hilden, Germany) from the bacterial colonies grown on blood agar plates. The Nextera XT DNA Library Preparation Kit (Illumina, Inc., San Diego, CA, USA) was used to prepare a sequencing library. The sequencing was performed as paired 2 × 150-bp reads on the NextSeq 500 (Illumina, Inc., San Diego, CA, USA). The raw reads were processed by the Tormes 1.3 pipeline (Quijada et al. 2019). The presence of exfoliative toxin genes in the raw genomes was investigated by aligning the nucleotide sequence of each gene deposited in the GenBank using the web-based Nucleotide Blast search tool (https://blast.ncbi.nlm.nih.gov/blast/Blast.cgi; accessed on 18 August 2025) (Camacho et al. 2009). The searched toxins and their accession numbers were as follows: toxin A ExhA (Accession No. AF515453), toxin B ExhB (Accession No. AF515454), toxin C ExhC (Accession No. AF515455), toxin D ExhD (Accession No. AF515456), exotoxin SHETA (Accession No. AB036768) and exotoxin SHETB (Accession No. AB036767) (Watanabe et al. 2000; Ahrens and Andresen 2004). The cut-off value for the presence of toxins was set at a minimum of 75% coverage of the database’s toxin genes and a percent identity of at least 90%.

### Detection of antimicrobial resistance

Antimicrobial resistance (AMR) was assessed by determining the minimum inhibitory concentrations (MICs) of twelve selected antimicrobials and one combination of antimicrobials using the microdilution method. The MICs were determined using diagnostic sets made at the VRI and the quality control of the MIC determination was assessed by the parallel examination of the control reference strain *Staphylococcus aureus* CCM 4223 (CLSI 2013). Genes of AMR (AGR; antimicrobial gene resistance) were determined by screening the whole genome of the isolates against the ResFinder (Zankari et al. 2012), CARD (McArthur et al. 2013) and ARG-ANNOT (Gupta et al. 2014) databases by using Abricate (Seemann 2025) within the TORMES 1.3.0 pipeline.

### Animals

Six piglets of the hybrid Large White (50%) × Landrace (50%) were selected from litters of the same age and weights (around 15 kg) from a conventional farm in the Czech Republic with a good epizootiological history and transported to the experimental animal facility of the VRI at the age of 28 days. The piglets (three males and three females) were randomly housed individually in pens in a conventional housing system suitable for pigs (temperature 24–30 °C and humidity 51–62%). The pens were marked with the piglet’s number. Throughout the trial, the animals had free access to drinking water and were fed the feed mixture Weaning Pellets (De Heus a.s., Bučovice, Czech Republic)* ad libitum*. The acclimatisation period lasted 7 days after the piglets’ arrival. All animal experiments were carried out in strict accordance with the Czech guidelines for animal experimentation and were approved by the Branch Commission for Animal Welfare of the Ministry of Agriculture of the Czech Republic (Approval Protocol No. MZe 2396).

### Preparation of the bacteria and experimental infection of the animals

Based on the results of the presence of toxin-encoding genes and antimicrobial resistance (described in detail below in the chapter Results and Discussion), three strains of *S. hyicus* were used: CAPM 6346, CAPM 6689, and CAPM 6690. All the strains were revitalised from freezing by inoculation on Columbia agar (Oxoid, Basingstoke, United Kingdom) with 5% of defibrinated sheep blood (blood agar) and incubation at 37 °C for 24 hours. Selected grown colonies from the blood agar were inoculated into a brain heart infusion (BHI) broth (Oxoid, Basingstoke, United Kingdom), and after 4 h of incubation at 37 °C on a shaker, the bacterial suspension was washed twice with sterile Dulbecco’s phosphate-buffered saline (D-PBS) (Capricorn Scientific, Ebsdorfergrund, Germany). The bacterial suspension was diluted to a final concentration of 1 × 10^8^ CFU/ml or 1 × 10^9^ CFU/ml. These concentrations were confirmed by tenfold dilutions plated on blood agar plates.

**Figure 1:**
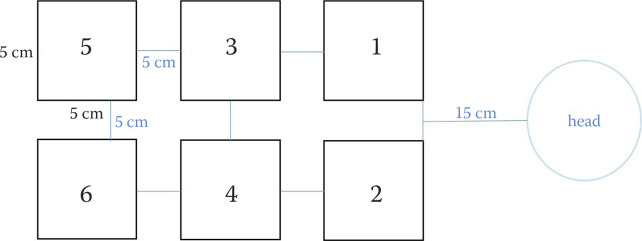
Scheme of the defect distribution in the piglets

On day 0 (D0 – the day after the end of the acclimatisation period), six superficial defects on the animal’s back in six places proximal to the neck and back after removal of the hair and antisepsis with a 2.5% povidone-iodine solution per pig were created by the scarification of a defect sized 5 × 5 cm. The scarification was conducted under a general injection anaesthesia (tiletamine 2 mg/kg + zolazepam 2 mg/kg (Zoletil 100; Virbac, Carlos, France) + ketamine 2 mg/kg (Narkamon; Bioveta, Ivanovice na Hané, Czech Republic) + xylazine 2 mg/kg (Xylazin Ecuphar 20 mg/ml; WERFFT, spol. s r. o., Brno, Czech Republic) prolonged with a general long-term inhalation anaesthesia with isoflurin 1 000 mg/g (Vetpharma Animal Health, S.L., Barcelona, Spain) at a concentration of 1–3% as follows: with a scalpel, the epidermis was removed in the given extent of the defect, and then with an 18G needle, ten parallel shallow incisions were made and then ten perpendicular lines in the shape of a grid were made. Then, the defects were inoculated with 3 ml of the bacterial culture at a concentration of 1 × 10^8^ CFU/ml (3 pigs) or 1 × 10^9^ CFU/ml (3 pigs), and covered with gauze. The defects were covered with several layers (sterile gauze, cover film, and an elastic Omnifix patch). The total wound coverage was protected from contamination and mechanical abrasion by a protective surgical gown. On every pig, three field strains (CAPM 6346, CAPM 6689 and CAPM 6690) were tested. The distribution of defects inoculated with the strains differed in every two piglets and is shown in Table 1 and Figure 1. The different distribution was intended to prevent the healing effect from depending on the location on the back (due to variations in skin thickness and properties). Analgesic medication was administered subcutaneously perioperatively using a Butomidor inj. (Richter Pharma AG, Wels, Austria) in a dose of 0.1 mg/kg body weight. After the creation and infection of defects, the analgesic Loxicom (meloxicam; Norbrook Laboratories, Newry, UK) was administered subcutaneously at a dose of 0.1 mg/kg body weight once daily for 3 consecutive days.

**Table 1:** Distribution of the *S. hyicus* strains on the defects of every pig

No. of defect	Strain of *S. hyicus*						
	concentration 1 × 10^8^ CFU/ml				concentration 1 × 10^9^ CFU/ml		
	pig 1	pig 2	pig 3		pig 4	pig 5	pig 6
1	CAPM 6346	CAPM 6690	CAPM 6689		CAPM 6346	CAPM 6690	CAPM 6689
2	CAPM 6346	CAPM 6690	CAPM 6689		CAPM 6346	CAPM 6690	CAPM 6689
3	CAPM 6689	CAPM 6346	CAPM 6690		CAPM 6689	CAPM 6346	CAPM 6690
4	CAPM 6689	CAPM 6346	CAPM 6690		CAPM 6689	CAPM 6346	CAPM 6690
5	CAPM 6690	CAPM 6689	CAPM 6346		CAPM 6690	CAPM 6689	CAPM 6346
6	CAPM 6690	CAPM 6689	CAPM 6346		CAPM 6690	CAPM 6689	CAPM 6346

Every day throughout the experiment, the pigs were clinically monitored, and their rectal temperature was measured. The piglets were monitored daily, and any changes in their general health were recorded. Changes in the activity, behaviour and attitude of the animals (pigs active, humped, slightly apathetic, markedly apathetic), breathing (rapid breathing at rest, coughing when moving, coughing at rest) and other impairments (neurological symptoms, impairment of the locomotor system) or injuries and possible deaths of the animals were monitored.

On D4, D9, and D14, a visual evaluation of the wounds (presence of erythema, scabs, oedema, exudate) and indirect imprints of the defects was performed. Before making the imprints, the animals were put under general short-term inhalation anaesthesia with isoflurane 1 000 mg/g (Vetpharma Animal Health, S.L., Barcelona, Spain) at 1–3% on D4 and D9, or under general injection anaesthesia on D14.

At the end of the experiment, on D14, the animals were euthanised by an intravenous administration of the T61 preparation (Intervet International B.V., Boxmeer, Netherlands) according to the manufacturer’s instructions under general injection anaesthesia.

### Microbiological assessment of the infection development

Indirect imprints were performed by applying a 5 × 5 cm filter paper to the defects for 30 s, then placing the paper on blood agar plates. After the samples were transported to the laboratory, the imprints on the filter papers were re-imprinted on another blood agar for 30 seconds. The samples were incubated at 37 °C for 24 hours. The imprints were evaluated semi-quantitatively (0, +, +/++, ++, ++/+++, +++, +++/++++, ++++).

### Statistical analysis

The results of the microbiological evaluation were statistically evaluated using a one way analysis of variance (ANOVA)/Kruskal-Wallis test with a subsequent Dunn’s multiple comparisons test. All the calculations were performed using the software Prism^®^ v8.0 (GraphPad Software, Inc., USA).

## RESULTS AND DISCUSSION

### Characterisation of the bacteria

The results of the pattern-matching from the Biotyper software are expressed as a score ranging from 0.000 to 3.000. According to the manufacturer, a score ≥2.300 is recommended for a highly probable species identification, a score from 2.000 to 2.299 for secure genus identification and probable species, and a score from 1.700 to 1.999 for a probable genus identification. A score below 1.700 indicates no reliable identification.

Three strains (CAPM 6346, CAPM 6690 and CAPM 6692) were interpreted as probable species *Staphylococcus hyicus* and two strains (CAPM 6689 and CAPM 6691) were interpreted as probable genus identification when using the standard ethanol-formic acid preparation protocol. The better results were obtained with the modified 30-minute formic acid extraction method. All five strains had a score value between 2.000 and 2.299 and were interpreted as probable species identification (*S.* *hyicus*).

The score value was found to be species-dependent in the bacteria (Richter et al. 2012; Szabados et al. 2012; Zhu et al. 2015). Lower scores have been suggested as acceptable for species identification of clinically relevant staphylococci (Richter et al. 2012; Szabados et al. 2012).

The historical strain and four isolates were identified using a STAPHYtest 24 kit, with additional tests, as *S. hyicus* (identification score: 98.32–100%). One strain, STH 3/22 (CAPM 6691), was identified by an API^®^ Staph kit as *S.* *hyicus* (identification score of 97%); other cultures were evaluated only to the genus level as *Staphylococcus* sp. The results of the recommended additional tests cannot be written to the identification profile on the website; therefore, the resulting identification is less accurate.

The results of the additional tests are summarised in Table 2. Three strains (CAPM 6689, CAPM 6690 and CAPM 6691) were detected as coagulase positive and two strains (CAPM 6346 and CAPM 6692) were coagulase negative. All five strains were catalase-positive. The results of the other additional tests were negative. These results are in accordance with the species characteristics described in the Bergey’s Manual of Systematic Bacteriology (Schleifer and Bell 2009).

**Table 2:** Properties of the *S. hyicus* strains, determined with additional tests

Test	CAPM 6346	CAPM 6689	CAPM 6690	CAPM 6691	CAPM 6692
Coagulase – tube agglutination test 24 h	–	+	+	+	–
Clumping factor – slide agglutination test 24 h	–	–	–	–	–
Novobiocin resistance	–	–	–	–	–
Acetoin production	–	–	–	–	–
Pyrrolidonyl arylamidase	–	–	–	–	–
Cytochrome oxidase	–	–	–	–	–
Catalase	+	+	+	+	+
Pigment production	–	–	–	–	–

(+) = positive reaction; (–) = negative reaction; CAPM = the Collection of Animal Pathogenic Microorganisms

The toxins, their coverage, and percent identity for each *S.* *hyicus* strain are shown in Table 3. The exotoxin SHETA was identified in all the strains. Exfoliative toxin A (ExhA) was identified in the strains CAPM 6346 and CAPM 6689. Moreover, toxin B (ExhB) and toxin D (ExhD) were identified with approximately 50% nucleotide sequence coverage, with 89% (ExhB) and 70% (ExhD) identity in these strains (CAPM 6346 and CAPM 6689). Exfoliative toxin ExhA was the most present in the *S.* *hyicus* strains in the study of Leekitcharoenphon et al. (2016) where all the toxigenic strains encoded one of the exfoliative toxins ExhA, ExhB, ExhC, or ExhD. In the present study, only few strains possessed one of the exfoliative toxins, while all the strains possessed the exotoxin SHETA. The exotoxin encoding gene *sheta* together with the exfoliative toxin-encoding genes *exhD* and *exhC* were mainly found among strains isolated from the exudative epidermitis (Kanbar et al. 2008). The exotoxin encoding gene *shetb* was not present among the *S.* *hyicus* strains in the study of Futagawa-Saito et al. (2007) or Kanbar et al. (2008) as well as in the present study.

**Table 3:** Identification of the toxins in each *S. hyicus* strain based on the coverage and identity of the nucleotide sequences detected in these strains by whole-genome sequencing and reference sequences using the blast+ software

Strain of *S. hyicus* (CAPM)	Toxin A ExhA			Toxin B ExhB			Toxin C ExhC			Toxin D ExhD			Exotoxin SHETA			Exotoxin SHETB	
	query cover (%)	percent identity (%)		query cover (%)	percent identity (%)		query cover (%)	percent identity (%)		query cover (%)	percent identity (%)		query cover (%)	percent identity (%)		query cover (%)	percent identity (%)
6346	98	100.00		53	89.20		6	87.95		54	70.07		100	96.24		0	0
6689	84	100.00		51	89.20		7	89.41		54	70.07		90	95.76		14	85.23
6690	9	100.00		11	97.69		6	87.95		0	0		100	96.24		0	0
6691	13	100.00		1	92.86		4	81.94		0	0		100	97.83		0	0
6692	13	100.00		1	92.86		7	81.94		0	0		89	95.70		0	0

The cut-off value for the presence of toxins was set at a minimum of 75% coverage of the database’s toxin genes and a percent identity of at least 90%

CAPM = the Collection of Animal Pathogenic Microorganisms

The MIC results are shown in Table 4. Although the clinical breakpoints for *S. hyicus* or *Staphylococcus* spp. in swine are not described in CLSI (2024), Moreno et al. (2022) calculated the applied breakpoints to *S.* *hyicus* in their study. When these breakpoints were applied to our strains in this study, all the strains were resistant to clindamycin and some strains were resistant to penicillin, ampicillin, erythromycin or tetracycline (Table 4, resistance is highlighted in grey). The AMR was also confirmed by the presence of at least one of the AGRs encoding resistance to the corresponding antimicrobial (Table 5) [e.g., *blaZ* for penicillin or ampicillin, *erm(A)* for erythromycin or clindamycin, *tet(M)* or *tet(K)* for tetracyclines], although not in all the strains (e.g., clindamycin, erythromycin). Genes encoding resistance to aminoglycoside antimicrobials were detected in two strains, though no resistance to gentamicin was detected by the microdilution method. Additionally, strain CAPM 6690 was intermediate to florfenicol and carried the *cat(pC233)* gene encoding chloramphenicol resistance, which may indicate possible resistance to phenicols. Multi-drug resistance (resistant to three or more different antimicrobial classes) was detected in two strains (CAPM 6689 and CAPM 6692). The other three strains were resistant or intermediate to two different antibiotic classes. In the study by Yun et al. (2024), all toxigenic strains were resistant to clindamycin, as was the case in our study. Erythromycin resistance in this bacterium was found to vary from 15% to 62% in Denmark (Wegener et al. 1994; Aarestrup and Jensen 2002). The resistance of *S.* *hyicus* to tetracycline has been detected in many other countries worldwide (Wegener et al. 1994; Park et al. 2013a; Moreno et al. 2022; Mencia-Ares et al. 2024; Yun et al. 2024). Relatively low levels of susceptibility to penicillin, ampicillin and florfenicol were also detected in *S.* *hyicus* strains isolated in Brazil (Moreno et al. 2022), South Korea (Yun et al. 2024) or Spain (Mencia-Ares et al. 2024). All our strains were susceptible to ceftiofur, a combination of trimethoprim/sulfamethoxazole, fluoroquinolones (enrofloxacin), rifaximin and penicillinase stable-penicillins (cefoxitin, oxacillin), which is consistent with the results of the study of Moreno et al. (2022) and of Yun et al. (2024), with the exception of enrofloxacin, which showed very low susceptibility in these studies. According to the Guidelines for the prudent use of antimicrobials in veterinary medicine (2015/C 299/04), antimicrobials from category B (enrofloxacin and ceftiofur) should only be used if there is no clinically effective alternative in categories C or D and based on bacterial susceptibility testing (European Commission 2015). According to this guideline, antimicrobials from category D (penicillin, ampicillin, tetracycline, trimethoprim, sulfamethoxazole, oxacillin in this study) are the first-line therapy in veterinary medicine while antimicrobials from category C (gentamicin, erythromycin, clindamycin, florfenicol in this study) are also alternatives in human medicine, so they should be used only if there is no clinically effective alternative in category D. For example, in the Czech Republic, the most commonly used antibiotics for treatment of exudative epidermitis include tetracyclines, gentamicin, trimethoprim/sulfamethoxazole and amoxicillin/clavulanic acid. Additionally, the cefoxitin and oxacillin tested here are used to detect the methicillin-resistant *Staphylococcaceae* species for which this diagnostic test was designed.

**Table 4:** Results of the MICs (minimum inhibition concentrations, μg/ml) of each *S.* *hyicus* strain

Antimicrobial class or subclass	Antimicrobial	Strain of *S. hyicus*				
		CAPM 6346	CAPM 6689	CAPM 6690	CAPM 6691	CAPM 6692
Aminoglycosides	gentamicin	≤0.25	≤0.25	≤0.25	≤0.25	≤0.25
						
Penicillinase labile-penicillins	penicillin	≤0.06	0.25	≤0.06	0.25	1
	ampicillin	0.25	1	0.25	1	4
	ceftiofur	0.5	0.5	0.5	0.5	1
						
Macrolides	erythromycin	>16	>16	0.25	≤0.125	≤0.125
Lincosamides	clindamycin	16	16	16	16	16
Phenicols	florfenicol	2	1	4	1	2
Tetracyclines	tetracycline	≤0.25	32	≤0.25	≤0.25	2
Sulfonamides	SXT^a^	0.125	0.125	0.125	0.125	0.125
Fluoroquinolones	enrofloxacin	0.125	≤0.06	0.125	0.125	0.125
Rifamycins	rifaximin	≤0.03	≤0.03	≤0.03	≤0.03	≤0.03
						
Penicillinase stable-penicillins	cefoxitin	≤4	≤4	≤4	≤4	≤4
	oxacillin	≤0.25	≤0.25	≤0.25	≤0.25	≤0.25

The MIC values indicating potential resistance and potential intermediates are shown in grey and pale grey, respectively

^a^SXT – trimethoprim/sulfamethoxazole, and its concentration is 1/19; the concentration given in the table refers to trimethoprim

**Table 5:** Antimicrobial resistance genes (ARGs) detected in each *S.* *hyicus* strain by Resfinder, CARD or ARG-ANNOT

Antimicrobial class	ARG	CAPM 6346	CAPM 6689	CAPM 6690	CAPM 6691	CAPM 6692
Aminoglycoside	*ant(9)-Ia*	0	1	0	0	0
	*str*	1	0	0	0	0
Penicillin	*blaZ*	0	1	0	1	1
Macrolides, lincosamide	*erm(A)*	0	1	0	0	0
						
Lincosamide	*lnu(B)*	0	0	0	0	1
	*lsa(E)*	0	0	0	0	1
	*vga(E)*	0	1	0	0	0
	*vga(A)LC*	0	0	1	0	0
						
Phenicols (chloramphenicol)	*cat(pC233)*	0	0	1	0	0
Tetracyclines	*tet(M)*	0	1	0	0	0
	*tet(K)*	0	0	0	0	1
ARG in total per strain		1	5	2	1	4

The presence of the gene is annotated as “1” and the absence of the gene is annotated as “0”. Resistance to the antimicrobials detected by the microdilution method and the presence of the corresponding genes of the individual strains are shown in grey

Based on the results of the toxin gene identification, the strains CAPM 6346, CAPM 6689, and CAPM 6690 were chosen to establish an *in* *vivo* infection model for *S.* *hyicus*. The first two strains carry genes encoding toxins ExhA and SHETA, possibly also toxin B and toxin D, and the third strain carries the *sheta* gene. Moreover, although the coverage of the nucleotide sequences of genes for toxin B and C in this strain was very low, it was higher than in the strains CAPM 6691 or CAPM 6692. That is the reason why we also chose the strain CAPM 6690.

### Experimental challenge

**Figure 2:**
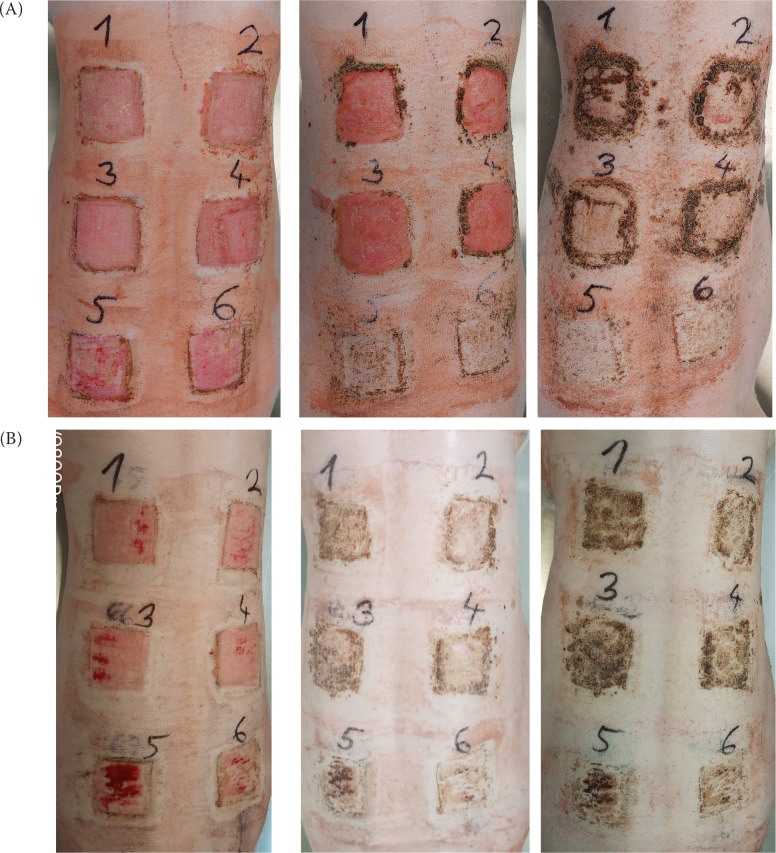
Healing of defects on various days post infection Distribution *of S.* *hyicus* strains: defect 1, 2: CAPM 6346; defect 3, 4: CAPM 6689; defect 5, 6: CAPM 6690 (A) Concentration of bacteria 10^8^ CFU/ml; (B) Concentration of bacteria 10^9^ CFU/ml

Every day for the duration of the experiment, the pigs were monitored, and the rectal temperature was measured for four days post-infection. The rectal temperature was under 40.5 °C and no difference was seen among the animals. On D4, D9, and D14, the defects were visually monitored for signs of inflammation, erythema, crusts, oedema, or exudate. An example of the course of wound healing is shown in Figure 2. It seems that a lower bacterial concentration led to lower secretion and the formation of crusts. On the other hand, the dermal/epidermal distribution of *S.* *hyicus* was observed on the back and bottom in one pig (cultivation confirmation) challenged with the lower concentration.

**Figure 3:**
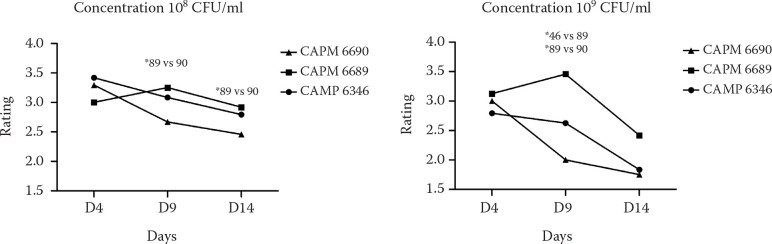
Bacterial evaluation of the wounds Data are expressed as the means of the semi-quantitative evaluation (+ = 1 point, +/++ = 1.5 points, ++ = 2 points, ++/+++ = 2.5 points, +++ = 3 points, +++/++++ = 3.5 points, ++++ = 4 points) of the imprints from six wound defects infected with the same strain and concentration. *The significant difference (*P *< 0.05) is denoted by an asterisk and with the groups, where 46 is the strain CAPM 6346, 89 is the strain CAPM 6689 and 90 is the strain CAPM 6690

Moreover, based on bacterial cultivation of the indirect imprints, *S.* *hyicus* was present in greater amounts in wound defects infected with the lower bacterial concentration, especially at the end of the experiment (Figure 3). Additionally, higher concentrations of the strain CAPM 6689 were detected on D9 and D14 compared to the strain CAPM 6690, at 10^8^ CFU/ml. In the concentration of 10^9^ CFU/ml, higher amounts of bacteria were determined in the imprints of the wounds infected with the strain CAPM 6689 in comparison to the wounds infected with the strain CAPM 6346 or with the strain CAPM 6690.

Overall, the strain CAPM 6689 at the concentration of 10^8^ CFU/ml was evaluated as the most suitable for the local infection development. This strain possesses the exotoxin SHETA, the exfoliative toxin ExhA, and likely ExhB and ExhD as well. In the study of Fudaba et al. (2005), each isoform of the exfoliative exotoxins (ExhA, ExhB, ExhC or ExhD) injected subcutaneously in piglets caused superficial epidermal blisters or crust formation similar to that observed in the pigs with exudative epidermitis. These authors confirmed that the exfoliative exotoxins of *S.* *hyicus* selectively degrade porcine desmoglein 1, a desmosomal intercellular adhesion molecule, thereby facilitating percutaneous bacterial invasion of the skin and leading to the exfoliation observed in the present study. Other isoforms of exfoliative toxins are also produced by virulent *Staphylococcus* strains, such as *S. aureus* and *S.* *chromogenes*, that cause loss of keratinocyte – cell adhesion in the superficial epidermis in a mammalian species-specific manner (Andresen et al. 2005; Nishifuji et al. 2005). Kanbar et al. (2008) suggested that the exotoxin SHETA also contributes to the clinical signs of exudative epidermitis. Our results confirmed this hypothesis. An experimental challenge with *S.* *hyicus* has been previously performed in mice and piglets intraperitoneally, intramuscularly, subcutaneously and by its application on full-thickness excision wounds, but not on the superficial excision (of epidermis) wounds (Svedman et al. 1989; Sato et al. 1990; Wang et al. 2016; Li et al. 2021; Liu et al. 2021; Ma et al. 2021). As *S.* *hyicus* is a commensal bacterium on a piglet’s skin, the main infection route is the disruption of the epidermal barrier caused by the teeth when suckling or weaning fighting in piglets (Wegener et al. 1993).

Thus, the experimental infection model presented in this study mimics the natural route of infection by epidermis scarification and deep incisions. The subsequent development of exudative epidermitis is very similar to that under field conditions. Therefore, this infection model is more suitable for following the testing of a local antimicrobial therapy than other infection routes*.*
